# Investigation on the role of VEGF gene polymorphisms in the risk of osteosarcoma

**DOI:** 10.12669/pjms.312.6533

**Published:** 2015

**Authors:** Zhao Li-lian, Wang Lin, Shi Lei, Zhang Yao-nan

**Affiliations:** 1 Zhao Li-lian, Department of Orthopedics, Beijing Hospital, Beijing 100730, China; 2 Wang Lin, Department of Orthopedics, Beijing Hospital, Beijing 100730, China; 3 Shi Lei, Department of Orthopedics, Beijing Hospital, Beijing 100730, China; 4 Zhang Yao-nan, Department of Orthopedics, Beijing Hospital, Beijing 100730, China

**Keywords:** Single nucleotide polymorphism, Vascular endothelial growth factor, Osteosarcoma

## Abstract

**Objective:**

The VEGF in low oxygen conditions are reported to prolong the survival of malignant cell, and thus this gene has a critical role in tumor growth and invasion as well as development of malignant tumor. We aimed to assess the association between the six common SNPs and the risk of osteosarcoma, and their association with environmental factors.

**Methods:**

176 subjects with osteosarcoma and 176 gender- and sex-matched healthy control individuals were enrolled into our study. The VEGF -2578C/A, -1156G/A, +1612G/A, +936C/T, -634G/C and -460T/C gene polymorphisms were determined using a polymerase chain reaction-restriction fragment length polymorphism (PCR-RFLP) assay according to manufacturer’s instructions

**Results:**

By conditional logistic regression analysis, AA and CA+AA genotypes of VEGF -2578C/A were associated with significant increased risk of osteosarcoma compared with CC genotype, and the ORs(95%CI) were 2.32(1.18-4.60) and 1.68(1.07-2.64), respectively. Moreover, individuals with CC and TC+CC genotypes of VEGF-460T/C had significant increased risk of osteosarcoma compared with those carrying with the TT genotype, and ORs(95%CI) were 2.15(1.10-4.21) and 1.60(1.0-2.58), respectively. By stratified analysis, we did not find statistically significant associated between VEGF -2578C/A and -460T/C gene polymorphisms and cancer risk by stratification analysis.

**Conclusion:**

Our results suggested that VEGF -2578C/A and -460T/C gene polymorphisms may be association with an increased risk of osteosarcoma.

## INTRODUCTION

Osteosarcoma is the most common type of primary bone cancer, and this cancer is a malignant tumor arising from mesenchymal tissues. Osteosarcoma mostly occurs in the long bones of the body, such as distal and proximal tibia and proximal humerus.^1^ This cancer often affects children, adolescents and young adults between 10 and 25 years of age, and often occurs in males than in females.^2^,^3^ Osteosarcoma is a kind of disease which is caused by complex, multistep, and multifactorial process. The exact etiology of osteosarcoma is not well understood, and previous studies have shown that many environmental and genetic factors are involved. It is well known that certain bone diseases and inherited cancer syndromes play an important role in the development of osteosarcoma.^4^-^6^ Previous molecular epidemiology studies showed that genetic polymorphisms are involved in the pathogenesis of osteosarcoma, such as growth and hormone genes and DNA repair genes as well as apoptosis and inflammatory genes.^7^-^9^

It is well known that vascular endothelial growth factors (VEGF), a potent angiogenic growth factor, plays an important role in altering proliferation to inflammatory and ischemic processes.^10^,^11^ The human VEGF is located at 6p21.1, and it is highly polymorphic in the promoter 5’untranslated region (5’-UTR) and 3’UTR.^12^-^14^ It is reported that single nucleotide polymorphisms (SNPs) in VEGF could regulate the expression of this gene through altering initiation of transcription and internal initiation of translation.^15^ Several SNPs in the 5’-UTR and 3’UTR were reported to be associated with alteration of VEGF protein production, including -2578C/A, -1156G/A, +1612G/A, +936C/T and -634G/C as well as -460T/C.

The VEGF in low oxygen conditions are reported to prolong the survival of malignant cell, and thus this gene has a critical role in tumor growth and invasion as well as development of malignant tumor.^11^ Only two studies have assessed the role of common VEGF polymorphisms in the risk of osteosarcoma in a Chinese population.^16^,^17^ Therefore, the aim of this present study was to assess the association between the six common SNPs and the risk of osteosarcoma, and their association with environmental factors.

## METHODS

### Study design and study populations

In the present hospital-based case-control study, 176 subjects with osteosarcoma and 176 gender- and sex-matched healthy control individuals were collected from the Department of Orthopedics of Beijing Hospital between May 2011 and December 2013. The osteosarcoma patients were newly diagnosed and histologically confirmed by two pathologists. The control subjects were randomly selected from a pool of individuals who were attending a clinic for routine examination during the same period. The controls were frequency matched to the cancer cases by age and gender, and the control subjects were free from any cancer or other chronic disease, and unrelated to the patients.

The written informed consent before participating into this study was obtained from all study participants, and this study was approved by the ethics committee of the Department of Orthopedics of Beijing Hospital.

The general demographic and clinical characteristics of osteosarcoma cases and control subjects were collected from medical records, including age, gender, family history of cancer, tumor location, therapy and stage of tumor.

### DNA extraction and genotyping

Peripheral venous blood was collected from each osteosarcoma case and control subject. According to the manufacturer’s instructions, genomic DNA was extarcted from collected peripheral blood mononuclear cells using TIANamp Blood DNA Kit (Tiangen Inc., Beijing, China). The VEGF -2578C/A, -1156G/A, +1612G/A, +936C/T, -634G/C and -460T/C gene polymorphisms were determined using a polymerase chain reaction-restriction fragment length polymorphism (PCR-RFLP) assay according to manufacturer’s instructions (Applied Biosystems, Foster City, CA, USA). The PCR primers of VEGF -2578C/A, -1156G/A, +1612G/A, +936C/T, -634G/C and -460T/C were designed by Sequenom Assay Design 3.1 software (Sequenom Inc., San Diego, CA). The PCR reaction conditions were as follows: 95 °C for one minute, then 40 cycles of 95 °C for 20 sec, 60 °C for 1 min and 72 °C for 1 min, and a final extension step at 72 °C for 10 min. The digested PCR products were visualized by ultraviolet (UV) light.

### Statistical analysis

Comparison of general demographic and clinical characteristics between groups was performed using χ^2^-test. The Hardy–Weinberg equilibriums of VEGF -2578C/A, -1156G/A, +1612G/A, +936C/T, -634G/C and -460T/C genotype frequencies in controls were tested using goodness-of-fit χ^2^-test. The odds ratio (OR) and their corresponding 95% confidence intervals (CI) for the association between the six VEGF gene polymorphisms and osteosarcoma risk were assessed by conditional logistic regression. Interaction between genotypes of the six VEGF gene polymorphisms and general demographic characteristics was assessed by conditional logistic regression. All *P*-values were two sided, and a *P*-value less than 0.05 was considered as statistical significant. All analyses were performed using SPSS version 16.0 statistical software (SPSS Inc, Chicago, IL).

## RESULTS

Selected demographic and clinical characteristics of the osteosarcoma patients and controls are shown in Table-I. Cases and controls did not differ regarding sex, age and family history of cancer (P>0.05). The mean age of osteosarcoma patients and control subjects were 18.4±11.5 years and 19.5±10.8 years old, respectively. Moreover, 76.14% of tumors located on long tubular bones and others on axial skeleton (23.86%). 57.39% of tumors were at I-II stage and others at III-IV stage (42.61%). 26.14% of patients received amputation and 73.86% received limb salvage therapy.

**Table-I:** Demographic and clinical characteristics of osteosarcoma cases and control subjects.

Variables	Osteosarcoma cases	%	Control subjects	%	χ2-test	P value
Age	18.4±11.5		19.5±10.8			
≤20	81	46.02	86	48.86		
>20	95	53.98	90	51.14	0.28	0.59
Gender						
Male	109	61.93	109	61.93		
Female	67	38.07	67	38.07	0.00	1.00
Family history of cancer						
No	16	9.09	13	7.39		
Yes	160	90.91	163	92.61	0.34	0.56
Tumor location						
Long tubular bones	134	76.14				
Axial skeleton	42	23.86				
Stage						
I-II	101	57.39				
III-IV	75	42.61				
Therapy						
Amputation	46	26.14				
Limb salvage	130	73.86				

The association of VEGF -2578C/A, -1156G/A, +1612G/A, +936C/T, -634G/C and -460T/C with risk of osteosarcoma are shown in Table-II. The genotype distributions of VEGF -2578C/A, -1156G/A, +1612G/A, -634G/C and -460T/C were not deviation from the expected Hardy–Weinberg equilibrium in the control subjects (*P*>0.05), but the genotype frequencies of +936C/T were not (*P*<0.05). By conditional logistic regression analysis, AA and CA+AA genotypes of VEGF -2578C/A were associated with significant increased risk of osteosarcoma compared with CC genotype, and the ORs(95%CI) were 2.32(1.18-4.60) and 1.68(1.07-2.64), respectively. Moreover, individuals with CC and TC+CC genotypes of -460T/C had significant increased cancer risk of osteosarcoma compared with those carrying with the TT genotype, and ORs(95%CI) were 2.15(1.10-4.21) and 1.60(1.0-2.58), respectively. However, no significant association was found between -1156G/A, +1612G/A, +936C/T and -634G/C and osteosarcoma risk in multivariate logistic regression analysis.

**Table-II:** Association between VEGF gene polymorphisms and risk of osteosarcoma.

SNPs	Genotype	Osteosarcoma group	%	Control group	%	OR(95%CI)^1^	P value
-2578C/A	CC	62	35.3	84	47.5	1.0(Ref.)	-
	CA	78	44.2	71	40.1	1.49(0.92-2.42)	0.09
	AA	36	20.5	21	12.4	2.32(1.18-4.60)	<0.05
	CA+AA	114	64.7	92	52.5	1.68(1.07-2.64)	<0.05
-1156G/A	AA	93	52.6	97	55.3	1.0(Ref.)	-
	AG	73	41.3	71	40.2	1.07(0.68-1.69)	0.75
	GG	11	6.1	8	4.5	1.43(0.50-4.30)	0.46
	AG+GG	84	47.4	79	44.7	1.11(0.71-1.72)	0.63
+1612G/A	CC	77	43.5	80	45.7	1.0(Ref.)	-
	CT	80	45.4	78	44.2	1.07(0.67-1.70)	0.78
	TT	19	11.1	18	10.1	1.10(0.50-2.40)	0.80
	CT+TT	99	56.5	96	54.3	1.07(0.69-1.67)	0.75
+936C/T	CC	85	48.2	92	52.4	1.0(Ref.)	-
	CT	75	42.3	71	40.1	1.14(0.72-1.82)	0.55
	TT	16	9.5	13	7.5	1.39(0.59-3.33)	0.41
	CT+TT	91	51.8	84	47.6	1.17(0.76-1.82)	0.46
-634G/C	CC	61	34.6	67	38.1	1.0(Ref.)	-
	CG	85	48.2	81	46.2	1.15(0.71-1.88)	0.55
	GG	30	17.2	28	15.7	1.18(0.60-2.30)	0.61
	CG+GG	115	65.4	109	61.9	1.16(0.73-1.83)	0.51
-460T/C	TT	48	28.4	66	37.3	1.0(Ref.)	-
	TC	89	50.3	85	48.2	1.44(0.87-2.39)	0.13
	CC	39	21.3	25	14.5	2.15(1.10-4.21)	<0.05
	TC+CC	128	71.6	110	62.7	1.60(1.0-2.58)	<0.05

1 Adjusted for sex, age and family history of cancer.

We assessed the association between VEGF -2578C/A and -460T/C gene polymorphisms and risk of osteosarcoma stratified by age, gender and family history of cancer (Table-III). However, we did not find statistically significant associated between VEGF -2578C/A and -460T/C gene polymorphisms and cancer risk after stratifying by age, gender and a family history of cancer.

**Table-III:** Stratification analysis on the association between VEGF -2578C/A and -460T/C and osteosarcoma risk by demographic characteristics

Variables	VEGF -2578C/A				OR(95%CI)^1^	P value	VEGF-460T/C				OR(95%CI)^1^	P value
	Cases		Controls				Cases		Controls			
	CC	CA+AA	CC	CA+AA	CA+AA vs CC		TT	TC+CC	TT	TC+CC	TC+CC vs TT	
*Age*												
≤20	27	54	39	47	1.66(0.85-3.27)	0.11	23	58	32	54	1.49(0.74-3.03)	0.23
>20	35	60	45	45	1.71(0.91-3.22)	0.07	25	70	34	56	1.7(0.87-3.34)	0.09
Gender												
Male	38	71	50	59	1.58(0.89-2.83)	0.1	30	79	41	68	1.59(0.86-2.93)	0.11
Female	24	43	34	33	1.97(0.93-4.20)	0.06	18	49	25	42	1.62(0.73-3.61)	0.2
*Family history of cancer*												
No	59	101	77	86	1.53(0.95-2.45)	0.06	44	116	61	102	1.58(0.96-2.60)	0.06
Yes	3	13	7	6	5.06(0.75-39.15)	0.05	4	12	5	8	1.87(0.29-12.49)	0.44

1 Adjusted for sex, age and family history of cancer.

## DISCUSSION

It is reported that many environmental and genetic factors can influence the individuals susceptibility to the carcinogenesis of osteosarcoma.^9^,^18^ Increasing evidences have reported that genetic polymorphisms play an important role in development of osteosarcoma, especially for angiogenesis genes.^4^-^6^ It is well known that angiogenesis plays an important role in the process of tumor growth, and can affect the tumor invasion and metastasis.^19^,^20^ The VEGF is highly polymorphic, and its polymorphisms are reported to influence the expression of VEGF.^15^

Several case-controls studies have reported the association of functional polymorphisms in VEGF with increased risk of several tumors, including osteosarcoma.^16^,^21^-^25^ Kapahi et al. conducted a case-control study to evaluate the association of seven VEGF promoter polymorphisms with breast cancer risk in a Indian population, and they found that VEGF-165C/T and -141A/C polymorphisms were association with decreased risk for breast cancer.^22^ Rinck-Junior et al. conducted a case-control study with 131 ovarian cancer patients and 137 controls, and found that VEGF +936C/T polymorphism increased the risk of ovarian cancer.^23^ Yang et al. investigated the role of three common VEGF polymorphisms in risk of bladder cancer, and did not find correlation between VEGF gene polymorphisms and bladder cancer risk.^24^ Chen et al. performed a meta-analysis with 5209 prostate cancer cases and 5233 controls, and found that no significant association between VEGF gene polymorphisms and risk of prostate cancer.^21^ Chen et al. also conducted a meta-analysis to explore the association between the VEGF -2578C/A polymorphism with the cancer risk, and they indicated that VEGF -2578C/A can influence the risk of colorectal cancer and lung cancer.^25^ The results of these studies are inconsistent. The discrepancies of these published results may be caused by differences in ethnicities, tumor types and study design as well as sample size.

For the association between VEGF gene polymorphisms and development of osteosarcoma, three previous studies have reported their association.^16^,^17^ Tie et al. assessed the association between VEGF gene polymorphisms and risk of osteosarcoma, and found that VEGF -2578C/A and -634G/C polymorphisms may influence the development of this cancer.^16^ Wang et al. also conducted a case-control study in a Chinese population, and suggested that +936C/T gene polymorphism had an important role in pathogenesis of osteosarcoma.^17^ In our study, we performed a case-control study to assess the role of six common gene polymorphisms in VEGF in the risk of osteosarcoma, and we found VEGF -2578C/A and -460T/C gene polymorphisms could affect the development of osteosarcoma. Therefore, further large sample studies are greatly needed to confirm the association of VEGF gene polymorphisms with osteosarcoma risk.

Two limitations should be considered in this study. First, the cases and controls were selected from on hospital, and distributions of VEGF +936C/T gene polymorphism in controls were not in line with Hardy–Weinberg equilibrium. The cases and controls may not represent the entire general population. Second, the small sample size of this study may limit the statistical power to find the difference between groups. Therefore, further studies with more different populations and larger sample size could help to verify the role of VEGF gene polymorphisms in the development of osteosarcoma.

In this case-control study, our results suggested that VEGF -2578C/A and -460T/C gene polymorphisms may be associated with an increased risk of osteosarcoma. Further large sample studies are greatly needed to confirm these associations.
